# Stress distribution and displacement in the maxillofacial complex during intrusion and distalization of the maxillary arch using miniplates versus mini-implants: a 3-dimensional finite element study

**DOI:** 10.1186/s40510-023-00455-6

**Published:** 2023-03-01

**Authors:** Abinaya Somaskandhan, N M. Vijay Kumar, R. Devaki Vijayalakshmi

**Affiliations:** Department of Orthodontic and Dentofacial Orthopaedics, Faculty of Dentistry, Meenakshi Ammal Dental College and Hospital, Maduravoyal, Chennai, Tamil Nadu India

**Keywords:** Finite element analysis, Miniplate, Mini-implant, Vertical maxillary excess, Distalization, Intrusion

## Abstract

**Objectives:**

To three-dimensionally analyse the stress distribution and displacement pattern in the maxillofacial complex following intrusion and distalization of the maxillary arch using finite element analysis in skeletal class II malocclusion with prognathic maxilla and vertical maxillary excess using miniplates and mini-implants.

**Materials and methods:**

Finite Element models of a skull, Y-shaped stainless steel miniplate, mini-implant and a posted arch were generated. Three force levels (1) 200 g (2) 300 g and (3) 500 g per side were applied to the assembly. The models were pre-processed and the analysis was performed using ANSYS version 18.1 software. Alterations in von mises stress, principal maximum stress, principal minimum stress and compressive stress were analysed around the sutures and surface landmarks.

**Results:**

With miniplates, there was a maximum stress concentration at the zygomatic buttress with even stress distribution at the fronto-maxillary, zygomatico-temporal, zygomatico-frontal and pterygomaxillary sutures along with anatomical landmarks such as frontal process of maxilla, ANS, Point A, prosthion and maxillary process of zygoma. First molars experienced greater distalization effects with buccal flaring when miniplates were used. With mini-implants, canine and premolars also exhibited greater distalization effects. In the root apices, lateral incisors showed increased lingual root movement with mini-implants.

**Conclusion:**

Miniplates provide a greater distalizing effect while mini-implants produce increased intrusive effect. The distalizing effect is greater when 500 g of force is applied using miniplates with significantly even stress distribution and displacement pattern.

## Introduction

Improvement of facial and smile aesthetics is of pre-eminent importance in orthodontic treatment. Aesthetic smile is defined as the relationship between the teeth, gingival scaffold and the lip framework [1]. Individuals with full maxillary incisor display along with a continuous band of gingiva are classified to have a high smile line commonly called as “gummy smile” and is one of the major reasons for patients seeking orthodontic treatment [2]. Management of vertical malocclusions of the face remains as one of the most challenging treatment procedures in orthodontics emphasizing accurate diagnosis and treatment planning [3]. Gummy smile can be an out-turn of profuse factors such as maxillary vertical excess, decreased incisor crown height, short/hyperactive upper lip and a combination of these conditions making the problem more challenging to treat [4].

During growth, orthopedic forces delivered using maxillary intrusion splint (MIS) assists in achieving good skeletal relationship thereby reducing the complexity of orthodontic treatment mechanics [5–9]. In adult patients, Le Fort I osteotomy with superior impaction is the most commonly used surgical procedure for correction of vertical maxillary excess [10]^.^

Miniplates and miniscrews are the anchorage systems that have revolutionized orthodontics [11, 12]. The literature available pertaining to the stress distribution and displacement pattern of miniplate and miniscrew implant is very sparse.

Hence, this study is aimed to three-dimensionally analyse the stress distribution and displacement pattern in the maxillofacial complex following intrusion and distalization of the maxillary arch using miniplates and mini-implants thereby enabling us to choose the best fit under suitable clinical circumstances.

## Materials and methods

### Construction of the finite element model

**Fig. 1:**
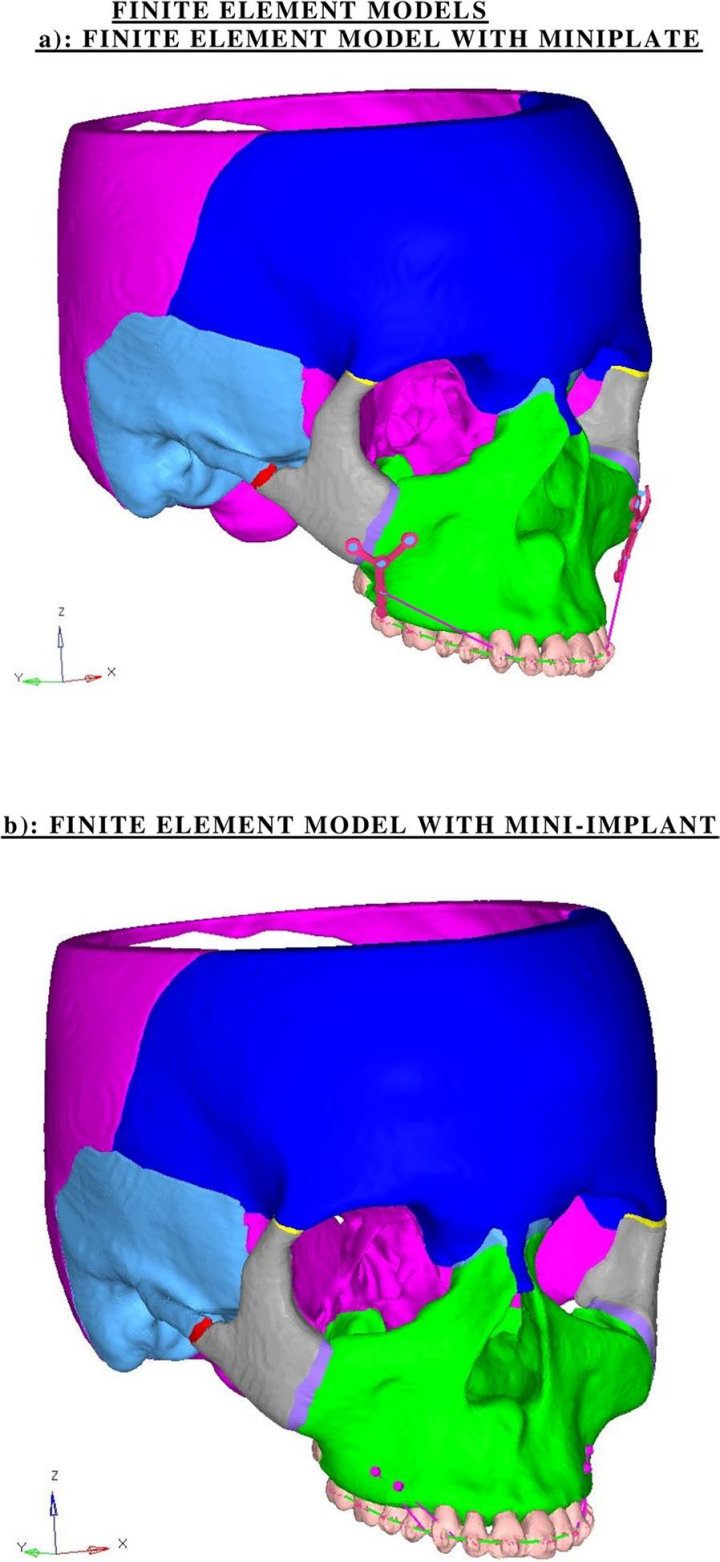
Finite element models. **a** Finite element model with miniplate. **b** Finite element model with mini-implant

A computer aided design model was created from the CT scan images of the skull of a patient with skeletal class II malocclusion with prognathic maxilla and vertical maxillary excess which were taken at 0.5 mm slice thickness. 3D models of the frontal bone, nasal bone, maxillary bone, zygomatic bone, temporal bone and sphenoid bone were generated individually. Sutures of the craniofacial complex were generated in the model with a width of 0.5 mm [13]. Teeth in the maxillary dentition were segmented and modelled individually. The periodontal ligament surrounding the maxillary teeth were modelled with a thickness of 0.2 mm [14]. DICOM images were generated and converted into STL file format using MIMICS software which were then assembled into a single unit and transferred to ANSYS software (Fig. 1).

3D model of a Y-type stainless steel miniplate and three mini-screws of dimension 1.5 × 8 mm to be threaded to fix the miniplate to the zygomatic buttress were generated for model 1. For model 2, two separate stainless steel mini-implants of size 1.5 × 8 mm were generated. One was placed in the interradicular space between the premolars at about 3 mm above the cementoenamel junction while the other was placed between the premolar and the molar at about 4 mm from the cementoenamel junction [15]. The variation in the height of the mini-implants were created in order to deliver a line of force which passes near the center of resistance of the maxillary arch.

Along with the facial bones, a total of five sutures namely the fronto-maxillary suture (FM), zygomatico-maxillary suture (ZM), zygomatico-temporal suture (ZT), zygomatico-frontal suture (ZF) and pterygomaxillary suture (PM) were analysed individually. Apart from the sutures, prime anatomical landmarks such as frontal process, anterior nasal spine, point A, prosthion and maxillary process of zygoma were evaluated separately. The material properties of all structures were assigned as shown in Table 1.

**Table 1:** Material properties

Material	Young’s modulus (MPa)	Poissons’s ratio
Cortical bone	13,700	0.30
Cancellous bone	7930	0.30
Miniplate	103,000	0.33
Miniscrew	10,300	0.33
Suture	68.65	0.40
Tooth	203,000	0.30
Stainless steel	2,059,000	0.30
Periodontal ligament	50.01	0.49

Forces applied were categorized into three levels. (1) 200 g per side, (2) 300 g per side and (3) 500 g per side. The force was applied 45° to the occlusal plane in order to achieve a line of force passing though the centre of resistance of the maxilla which is in the postero-superior aspect of the zygomatico-frontal suture.

### Boundary conditions

Foramen magnum was fixed as the origin point while the frontal bone was fixed as the upper limiting structure [16]. 3D co-ordinates were assigned as *X* plane indicating sagittal plane, *Z* plane indicating the vertical plane and *Y* plane indicating the transverse plane. Positive value in the *X* plane indicates a forward displacement, in *Z* plane indicates an upward displacement and in *Y* plane indicates an inward displacement.

## Results

### Illustration of the FE model

**Fig. 2:**
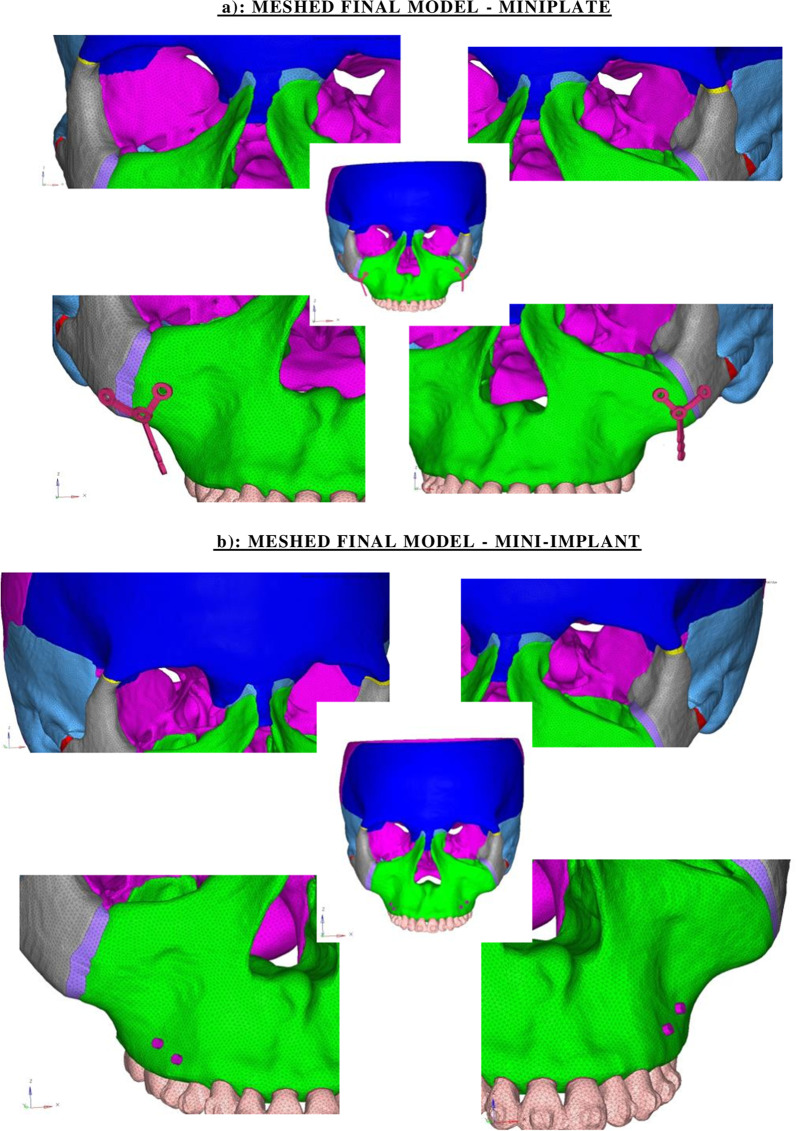
**a** Meshed final model—miniplate. **b** Meshed final model—mini-implant

**Fig. 3:**
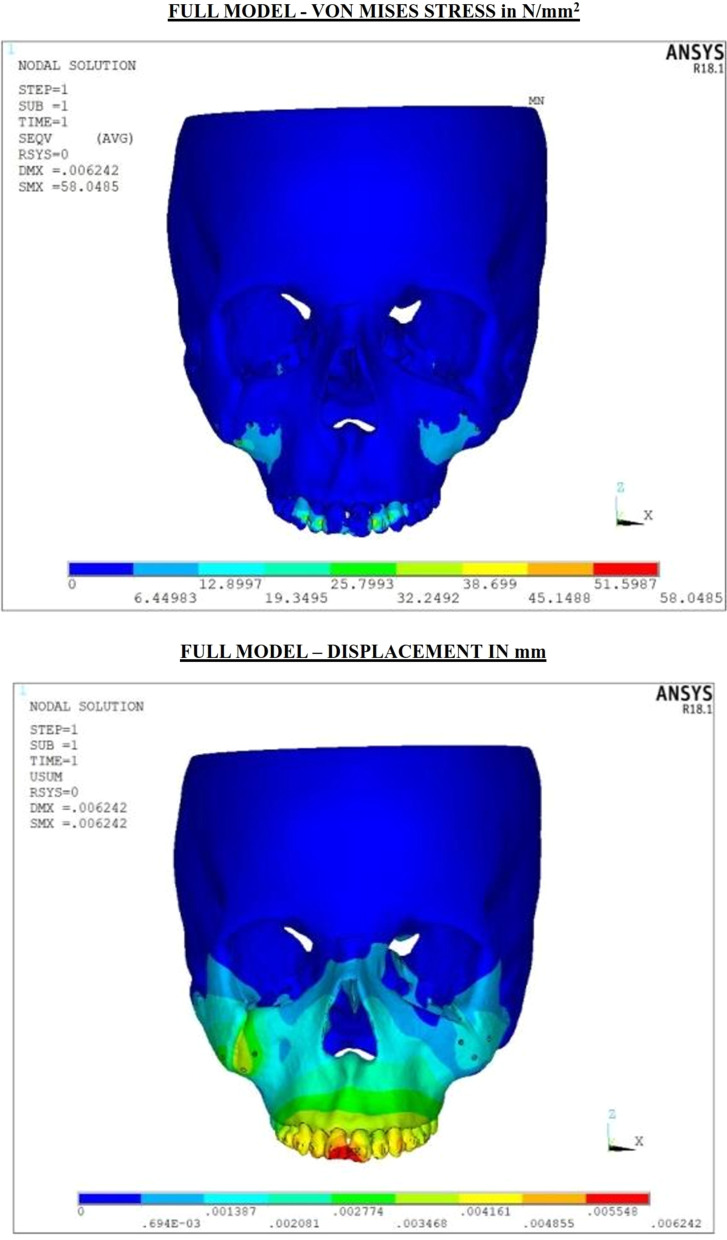
Stress and displacement plots in full model with miniplates for 500 g of force

**Fig. 4:**
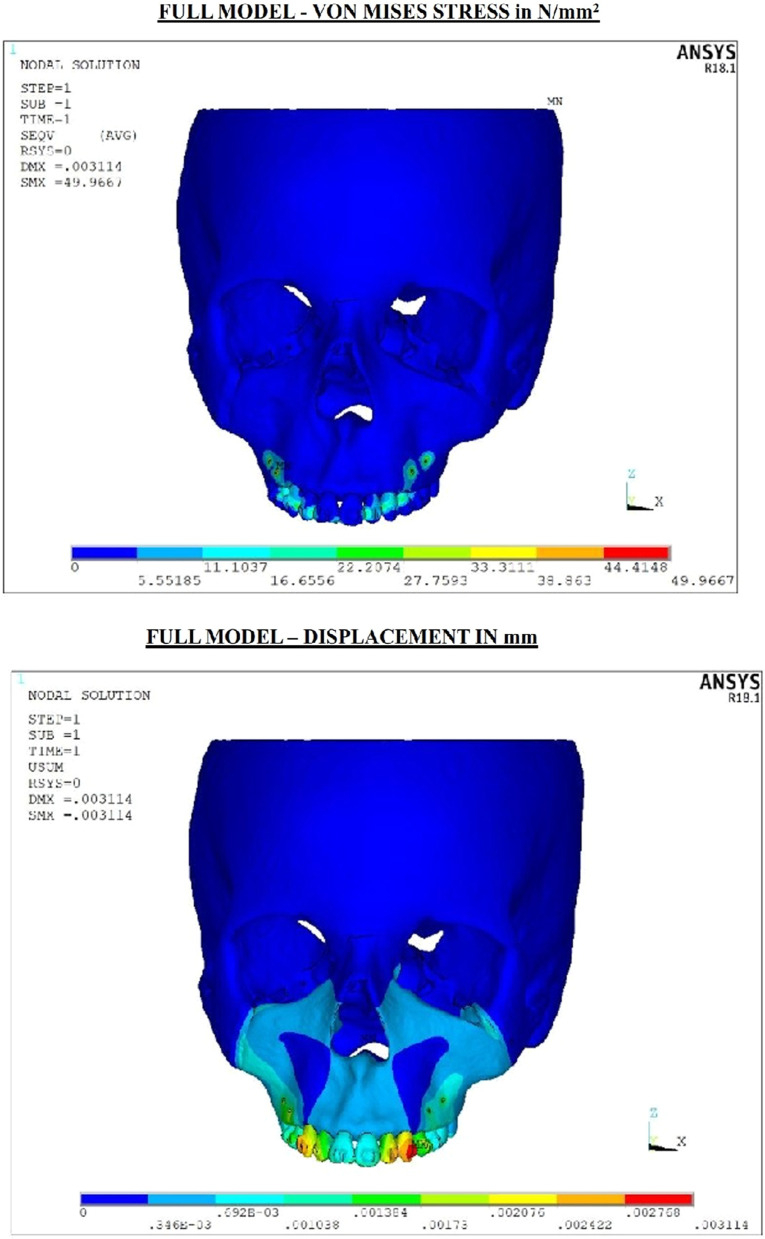
Stress and displacement plots in full model with mini-implants for 500 g of force

The final meshed model consisted of 298,551 nodes and 1,671,812 elements (Fig. 2). Alterations in the von mises stress, principal maximum stress, principal minimum stress and compressive stress were analyzed to compute the amount of stress distribution and displacement (Figs. 3, 4) (Tables 2a, b, 3).

**Table 2:** (a) Stress distribution and displacement in the sutures with miniplate, (b) stress distribution and displacement in the sutures with mini-implant

Appliance system	FM	TD–FM	*X* axis—FM	*Y* axis—FM	*Z* axis—FM	ZM	TD–ZM	*X* axis—ZM	*Y* axis—ZM	*Z* axis—ZM
1	0.11096	2.22E−04	4.42E−05	− 9.13E−05	1.87E−04	5.69E−02	1.55E−03	3.81E−05	1.24E−03	8.99E−04
1	0.012632	2.38E−04	5.46E−05	− 8.35E−05	2.13E−04	6.42E−02	1.68E−03	2.37E−04	1.39E−03	1.04E−03
1	0.014168	2.55E−04	6.49E−05	− 7.57E−05	2.39E−04	7.15E−02	1.80E−03	4.36E−04	1.54E−03	1.18E−03
1	6.99E−03	1.79E−04	1.63E−05	− 6.04E−05	1.40E−04	7.65E−02	7.70E−04	6.03E−04	5.61E−04	2.85E−04
1	7.95E−03	1.95E−04	2.59E−05	− 5.18E−05	1.57E−04	8.70E−02	8.38E−04	7.04E−04	6.43E−04	3.48E−04
1	8.91E−03	2.12E−04	3.55E−05	− 4.32E−05	1.75E−04	9.74E−02	9.06E−04	8.05E−04	7.24E−04	4.11E−04
2	1.59E−02	2.76E−04	8.92E−05	− 3.96E−05	2.56E−04	8.13E−02	2.32E−03	4.38E−05	1.90E−03	1.30E−03
2	1.81E−02	3.02E−04	1.02E−04	− 2.35E−05	2.91E−04	9.17E−02	2.50E−03	3.41E−04	2.12E−03	1.51E−03
2	2.03E−02	3.29E−04	1.14E−04	− 1.15E−05	3.25E−04	1.02E−01	2.69E−03	6.38E−04	2.35E−03	1.71E−03
2	1.19E−02	2.68E−04	2.45E−05	− 9.05E−05	2.09E−04	1.17E−01	1.42E−03	1.04E−03	1.14E−03	4.24E−04
2	1.35E−02	2.93E−04	3.89E−05	− 7.77E−05	2.36E−04	1.33E−01	1.56E−03	1.20E−03	1.30E−03	5.07E−04
2	1.52E−01	3.18E−04	5.33E−05	− 6.48E−05	2.63E−04	1.49E−01	1.71E−03	1.35E−03	1.46E−03	5.91E−04
3	2.66E−01	6.70E−04	8.89E−05	− 3.87E−04	4.80E−04	1.41E−01	3.62E−03	1.52E−04	2.85E−03	2.20E−03
3	3.03E−02	7.13E−04	1.14E−04	− 3.68E−04	5.48E−04	1.51E+05	3.93E−03	6.38E−04	3.20E−03	2.54E−03
3	3.40E−02	7.56E−04	1.40E−04	− 3.50E−04	6.17E−04	1.78E−01	4.24E−03	1.12E−03	3.56E−03	2.88E−03
3	1.88E−02	6.73E−04	− 3.53E−05	− 3.84E−04	3.40E−04	1.86E−01	1.75E−03	1.46E−03	1.09E−03	7.43E−04
3	2.14E−02	7.22E−04	− 1.38E−05	− 3.44E−04	3.85E−04	2.11E−01	1.90E−03	1.71E−03	1.26E−03	9.09E−04
3	2.41E−02	7.71E−04	7.72E−06	− 3.05E−04	4.31E−04	2.37E−01	2.05E−03	1.96E−03	1.42E−03	1.07E−03
4	4.59E−03	9.23E−05	6.16E−05	− 3.46E−05	− 5.51E−06	9.27E−03	2.66E−04	9.90E−05	3.43E−05	2.51E−04
4	5.24E−03	1.02E−04	7.24E−05	− 3.07E−05	3.45E−06	1.05E−02	3.03E−04	1.17E−04	6.46E−05	2.86E−04
4	5.89E−03	1.11E−04	8.32E−05	− 2.67E−05	1.24E−05	1.18E−02	3.40E−04	1.36E−04	9.48E−05	3.22E−04
4	5.64E−03	9.13E−05	2.04E−05	5.22E−06	− 2.05E−05	1.54E−01	2.25E−04	− 3.20E−06	9.15E−05	2.02E−04
4	6.44E−03	1.04E−04	2.92E−05	1.23E−05	− 7.21E−06	1.73E−01	2.57E−04	2.19E−05	1.12E−04	2.31E−04
4	7.23E−03	1.16E−04	3.80E−05	1.94E−05	6.05E−06	1.93E−01	2.88E−04	4.70E−05	1.33E−04	2.61E−04
5	6.89E−03	1.38E−04	9.24E−05	− 5.19E−05	− 8.27E−06	1.39E−01	3.98E−04	1.48E−04	5.14E−05	3.76E−04
5	7.86E−03	1.52E−04	1.09E−04	− 4.60E−05	5.18E−06	1.58E−02	4.54E−04	1.76E−04	9.68E−05	4.30E−04
5	8.84E−03	1.67E−04	1.25E−04	− 4.01E−05	1.86E−05	1.77E−01	5.10E−04	2.04E−04	1.42E−04	4.84E−04
5	8.46E−03	1.37E−04	3.05E−05	7.83E−06	− 3.07E−05	2.30E−02	3.38E−04	− 4.80E−06	1.37E−04	3.03E−04
5	9.65E−03	1.56E−04	4.38E−05	1.85E−05	− 1.08E−05	2.60E−02	3.85E−04	3.28E−05	1.68E−04	3.47E−04
5	1.08E−02	1.74E−04	5.70E−05	2.91E−05	9.07E−06	2.89E−02	4.32E−04	7.04E−05	1.99E−04	3.91E−04
6	1.15E−02	2.31E−04	1.54E−04	− 8.65E−05	− 1.38E−05	2.32E−02	6.64E−04	2.47E−04	8.57E−05	6.26E−04
6	1.31E−02	2.54E−04	1.81E−04	− 7.66E−05	8.63E−06	2.63E−02	7.57E−04	2.93E−04	1.61E−04	7.16E−04
6	1.47E−02	2.78E−04	2.00E−04	− 6.68E−05	3.10E−05	2.95E−02	8.50E−04	3.39E−04	2.37E−04	8.06E−04
6	1.41E−02	2.28E−04	5.09E−05	1.30E−05	− 5.11E−05	3.84E−02	5.64E−04	− 8.00E−06	2.29E−04	5.05E−04
6	1.61E−02	2.59E−04	7.30E−05	3.08E−05	− 1.80E−05	4.33E−02	6.41E−04	5.47E−05	2.80E−04	5.79E−04
6	1.81E−02	2.90E−04	9.50E−05	4.85E−05	1.51E−05	4.82E−02	7.19E−04	1.17E−04	3.32E−04	6.65E−04

Appliance system	ZT	TD–ZT	*X* axis—ZT	*Y* axis—ZT	*Z* axis—ZT	ZF	TD–ZF	*X* axis—ZF	*Y* axis—ZF	*Z* axis—ZF
1	9.45E−02	6.62E−04	− 3.46E−05	3.23E−04	4.87E−04	5.86E−03	1.12E−04	2.25E+03	5.58E−05	9.01E−05
1	1.08E−01	7.10E−04	1.15E−05	3.93E−04	5.17E−04	6.62E−03	1.23E−04	3.74E−05	6.03E−05	1.05E−04
1	1.21E−01	7.57E−04	5.77E−05	4.63E−04	5.47E−04	7.38E−03	1.34E−04	5.23E−05	6.48E−05	1.21E−04
1	1.85E−02	5.14E−04	3.13E−04	2.46E−04	3.35E−04	5.05E−03	1.78E−04	7.07E−05	8.68E−05	1.51E−04
1	1.99E−02	5.68E−04	3.67E−04	2.97E−04	3.69E−04	5.64E−03	2.00E−04	8.90E−05	9.74E−05	1.72E−04
1	2.13E−02	6.22E−04	4.21E−04	3.47E−04	4.04E−04	6.23E−03	2.22E−04	1.07E−04	1.08E−04	1.94E−04
2	1.49E−01	1.03E−03	− 3.27E−05	5.14E−04	7.62E−04	8.95E−03	1.74E−04	2.00E−05	9.19E−05	1.42E−04
2	1.69E−01	1.11E−03	3.95E−05	6.25E−04	8.09E−04	1.01E−02	1.90E−04	4.23E−05	9.84E−05	1.65E−04
2	1.90E−01	1.18E−03	1.12E−04	7.36E−04	8.56E−04	1.13E−02	2.06E−04	6.47E−05	1.05E−04	1.88E−04
2	3.72E−02	1.00E−03	5.05E−04	5.56E−04	6.66E−04	8.26E−03	3.13E−04	1.69E−04	1.73E−04	2.53E−04
2	4.02E−02	1.10E−03	6.12E−04	6.66E−04	7.33E−04	9.93E−03	3.52E−04	2.04E−04	1.94E−04	2.88E−04
2	4.32E−02	1.21E−03	7.19E−04	7.77E−04	7.99E−04	1.03E−01	3.91E−04	2.38E−04	2.15E−04	3.22E−04
3	2.05E−01	1.47E−03	− 1.40E−04	6.94E−04	1.07E−03	1.41E−02	2.66E−04	7.82E−05	1.25E−04	2.12E−04
3	2.34E−01	1.58E−03	− 3.83E−05	8.45E−04	1.14E−03	1.60E−02	2.94E−04	1.44E−04	1.36E−04	2.48E−04
3	2.63E−01	1.69E−03	6.36E−05	9.97E−04	1.21E−03	1.78E−02	3.22E−04	1.50E−04	1.47E−04	2.84E−04
3	3.89E−02	1.13E−03	7.87E−04	4.42E−04	7.15E−04	1.28E−01	4.31E−04	1.20E−04	1.85E−04	3.76E−04
3	4.18E−02	1.25E−03	9.05E−04	5.40E−04	7.89E−04	1.42E−01	4.84E−04	1.62E−04	2.08E−04	4.31E−04
3	4.47E−02	1.37E−03	1.02E−03	6.39E−04	8.63E−04	1.56E−02	5.37E−04	2.03E−04	2.31E−04	4.86E−04
4	2.20E−03	1.36E−05	1.09E−05	4.96E−06	7.22E−06	4.90E−05	7.05E−06	6.98E−06	1.49E−06	− 2.18E−07
4	2.51E−03	1.45E−05	1.16E−05	6.63E−06	7.64E−06	5.51E−05	7.45E−06	7.38E−06	1.72E−06	− 7.93E−08
4	2.82E−03	1.53E−05	1.24E−05	8.29E−06	8.06E−06	6.12E−05	7.85E−06	7.77E−06	1.96E−06	5.91E−08
4	1.15E−03	3.98E−05	7.90E−06	1.96E−05	3.10E−05	2.80E−04	1.59E−05	− 8.50E−06	6.77E−06	8.06E−06
4	1.65E−03	4.32E−05	1.22E−05	2.42E−05	3.37E−05	3.09E−04	1.70E−05	− 7.62E−06	7.26E−06	9.25E−06
4	1.78E−03	4.67E−05	1.66E−05	2.88E−05	3.64E−05	3.37E−04	1.80E−05	− 6.74E−06	7.76E−06	1.04E−05
5	3.30E−03	2.05E−05	1.64E−05	7.44E−06	1.08E−05	7.35E−05	1.06E−05	1.05E−05	2.24E−06	− 3.27E−07
5	3.76E−03	2.17E−05	1.75E−05	9.94E−06	1.15E−05	8.26E−05	1.12E−05	1.11E−05	2.59E−06	− 1.19E−07
5	4.22E−03	2.29E−05	1.86E−05	1.24E−05	1.21E−05	9.17E−05	1.18E−05	1.17E−05	2.94E−06	8.86E−08
5	2.28E−03	5.96E−05	1.19E−05	2.95E−05	4.66E−05	4.20E−04	2.39E−05	− 1.28E−05	1.02E−05	1.21E−05
5	2.48E−03	6.48E−05	1.84E−05	3.63E−05	5.06E−05	4.63E−04	2.54E−05	− 1.14E−05	1.09E−05	1.39E−05
5	2.68E−03	7.00E−05	2.49E−05	4.31E−05	5.46E−05	5.06E−04	2.69E−05	− 1.01E−05	1.16E−05	1.57E−05
6	5.50E−03	3.41E−05	2.73E−05	1.24E−05	1.80E−05	1.22E−04	1.76E−05	1.74E−05	3.73E−06	− 5.44E−07
6	6.27E−03	3.61E−05	2.91E−05	1.66E−05	1.91E−05	1.38E−04	1.86E−05	1.84E−05	4.31E−06	− 1.98E−07
6	7.04E−03	3.82E−05	3.09E−05	2.07E−05	2.02E−05	1.53E−04	1.96E−05	1.94E−05	4.89E−06	− 1.48E−07
6	3.80E−03	9.94E−05	1.98E−05	4.91E−05	7.76E−05	7.01E−04	3.99E−05	− 2.13E−05	1.69E−05	2.01E−05
6	4.13E−03	1.08E−04	3.06E−05	6.05E−05	8.43E−05	7.72E−04	4.24E−05	− 1.91E−05	1.82E−05	2.31E−05
6	4.46E−03	1.17E−04	4.14E−05	7.19E−05	9.10E−05	8.43E−04	4.49E−05	− 1.68E−05	1.94E−05	2.61E−05

Appliance system	PM	TD–PM	*X* axis—PM	*Y*axis—PM	*Z* axis—PM
1	3.06E−02	6.20E−04	4.20E−04	− 9.39E−06	− 1.56E−0
1	3.49E−02	6.91E−04	4.81E−04	5.24E−05	− 1.06E−04
1	3.92E−02	7.63E−04	5.42E−04	1.14E−04	− 5.49E−05
1	2.70E−02	7.30E−04	− 4.81E−05	− 1.65E−04	− 2.22E−04
1	3.08E−02	8.11E−04	− 1.36E−05	− 9.97E−05	− 1.68E−04
1	3.46E−02	8.92E−04	2.09E−05	− 3.46E−05	− 1.13E−04
2	2.04E+00	5.27E−04	3.95E−04	6.61E−05	− 2.14E−04
2	2.32E+00	5.77E−04	4.55E−04	1.15E−04	− 1.85E−04
2	2.60E+00	6.27E−04	5.15E−04	1.64E−04	− 1.56E−04
2	1.73E+00	5.11E−04	− 1.28E−04	− 1.33E−04	− 1.71E−04
2	1.97E+00	5.59E−04	− 8.79E−05	− 9.51E−05	− 1.47E−04
2	2.21E+00	6.06E−04	− 4.81E−05	− 5.68E−05	− 1.24E−04
3	5.60E−02	2.25E−03	1.32E−03	− 3.14E−04	− 8.26E−04
3	6.34E−02	2.48E−03	1.50E−03	− 1.03E−04	− 6.98E−04
3	7.09E−02	2.72E−03	1.69E−03	− 1.08E−04	− 5.70E−04
3	6.21E−02	2.45E−03	7.59E−05	− 6.24E−04	− 9.28E−04
3	7.06E−01	2.67E−03	1.94E−04	− 3.90E−04	− 7.97E−04
3	7.90E−02	2.89E−03	3.13E−04	− 1.56E−04	− 6.66E−04
4	3.96E−01	4.96E−05	4.55E−05	1.78E−05	4.28E−06
4	4.48E−01	5.38E−05	4.92E−05	2.26E−05	6.06E−06
4	5.01E−01	5.80E−05	5.30E−05	2.74E−05	7.83E−06
4	4.15E−01	2.98E−05	− 1.44E−05	1.34E−05	3.12E−06
4	4.70E−01	3.20E−05	− 1.22E−05	1.60E−05	5.59E−06
4	5.25E−01	3.41E−05	− 1.00E−05	1.86E−05	8.07E−06
5	5.94E−01	7.44E−05	6.82E−05	2.68E−05	6.42E−06
5	6.73E−01	8.07E−05	7.38E−05	3.39E−05	9.09E−06
5	7.51E−01	8.70E−05	7.95E−05	4.11E−05	1.18E−05
5	6.23E−01	4.47E−05	− 2.16E−05	2.00E−05	4.68E−06
5	7.05E−01	4.79E−05	− 1.83E−05	2.40E−05	8.39E−06
5	7.88E−01	5.12E−05	− 1.51E−05	2.80E−05	1.21E−05
6	9.90E−01	1.24E−04	1.14E−04	4.46E−05	1.07E−05
6	1.12E+00	1.34E−04	1.24E−04	5.65E−05	1.51E−05
6	1.25E+00	1.45E−04	1.32E−04	6.84E−05	1.96E−05
6	1.04E+00	7.44E−05	− 3.59E−05	3.34E−05	7.80E−06
6	1.18E+00	7.99E−05	− 3.05E−05	4.00E−05	1.40E−05
6	1.31E+00	8.54E−05	− 2.51E−05	4.66E−05	2.02E−05

*Average values of the right side and left side of each suture and the surface landmarks are taken

Appliance system 1—Miniplate—200 g of force, Appliance system 2—Miniplate—300 g of force, Appliance system 3—Miniplate—500 g of force, Appliance system 4—Mini-implant –200 g of force, Appliance system 5—Mini-implant—300 g of force, Appliance system 6—Mini-implant—500 g of force, *FM* fronto-maxillary suture, *ZM* zygomatico-maxillary suture, *ZT* zygomatico-temporal suture, *ZF* zygomatico-frontal suture, *PM* pterygo-maxillary suture, *TD* total displacement, *ANS* anterior nasal spine

**Table 3:** Stress distribution and displacement in anatomical landmarks

Appliance system	Frontal process	TD	*X*-axis	*Y*-axis	*Z*-axis	ANS	TD	*X*-axis	*Y*-axis	*Z*-axis
1	1.16529	3.51E−04	− 1.97E−05	− 1.07E−04	2.33E−04	5.69E−01	8.52E−04	− 3.01E−04	− 4.40E−04	4.88E−04
1	1.31896	3.80E−04	1.77E−05	− 1.02E−04	2.55E−04	6.44E−01	8.91E−04	− 2.84E−04	− 4.05E−04	5.23E−04
1	1.47264	4.08E−04	5.51E−05	− 9.63E−05	2.78E−04	7.20E−01	9.31E−04	− 2.68E−04	− 3.69E−04	5.58E−04
1	5.12E−01	3.19E−04	3.19E−04	− 1.79E−04	1.90E−04	1.23E+00	8.69E−04	− 2.47E−04	− 4.54E−04	4.82E−04
1	5.85E−01	3.42E−04	3.42E−04	− 1.64E−04	2.09E−04	1.40E+00	9.11E−04	− 2.22E−04	− 4.15E−04	5.20E−04
1	6.57E−01	3.64E−04	3.64E−04	− 1.49E−04	2.29E−04	1.57E+00	9.52E−04	− 1.96E−04	− 3.77E−04	5.58E−04
2	1.58E+00	3.36E−04	1.81E−05	− 2.69E−05	2.40E−04	8.22E−01	8.95E−04	− 3.27E−04	− 2.81E−04	6.48E−04
2	1.79E+00	3.69E−04	6.31E−05	− 1.60E−05	2.64E−04	9.31E−01	9.40E−04	− 3.06E−04	− 2.39E−04	6.90E−04
2	2.00E+00	4.03E−04	1.08E−04	− 5.12E−06	2.89E−04	1.04E+00	9.84E−04	− 2.85E−04	− 1.96E−01	7.32E−04
2	7.15E−01	2.94E−04	9.45E−05	− 1.17E−04	2.21E−04	1.80E+00	9.06E−04	− 2.36E−04	− 2.89E−04	6.40E−04
2	8.16E−01	3.17E−04	1.19E−04	− 1.04E−04	2.42E−04	2.05E+00	9.57E−04	− 2.05E−04	− 2.45E−04	6.86E−04
2	9.17E−01	3.40E−04	1.43E−04	− 9.06E−05	2.63E−04	2.30E+00	1.01E−03	− 1.74E−04	− 2.00E−04	7.32E−04
3	3.01E+00	1.11E−03	− 7.92E−05	− 4.91E−04	6.92E−04	1.50E+00	2.58E−03	− 7.53E−04	− 1.63E−03	1.29E−03
3	3.41E+00	1.19E−03	1.62E−05	− 4.68E−04	7.63E−04	1.70E+00	2.69E−03	− 7.11E−04	− 1.53E−03	1.39E−03
3	3.81E+00	1.27E−03	1.12E−04	− 4.45E−04	8.35E−04	1.90E+00	2.80E−03	− 6.68E−04	− 1.43E−03	1.49E−03
3	1.47E+00	1.10E−03	− 1.53E−05	− 6.37E−04	5.90E−04	3.13E+00	2.64E−03	− 6.11E−04	− 1.67E−03	1.28E−03
3	1.67E+00	1.17E−03	2.78E−05	− 5.82E−04	6.54E−04	3.57E+00	2.75E−03	− 5.45E−04	− 1.56E−03	1.38E−03
3	1.88E+00	1.25E−03	7.09E−05	− 5.27E−04	7.18E−04	4.00E+00	2.86E−03	− 4.79E−04	− 1.45E−03	1.49E−03
4	4.20E−01	8.59E−05	4.32E−05	− 4.50E−05	1.01E−05	3.84E−01	2.35E−04	− 1.95E−06	− 8.14E−06	− 8.80E−04
4	4.77E−01	9.21E−05	5.14E−05	− 3.00E−05	1.90E−05	4.36E−01	2.44E−04	4.42E−06	− 8.70E−07	− 1.79E−04
4	5.34E−01	9.83E−05	5.96E−05	− 3.48E−05	2.78E−05	4.88E−01	2.53E−04	1.08E−05	6.40E−06	− 1.70E−04
4	4.65E−01	1.01E−04	1.40E−05	− 3.91E−07	− 1.16E−05	5.40E−01	2.31E−04	− 1.43E−05	− 3.71E−06	− 1.73E−04
4	5.30E−01	1.11E−04	2.60E−05	9.50E−06	2.87E−06	6.15E−01	2.42E−04	− 6.69E−06	4.18E−06	− 1.62E−04
4	5.96E−01	1.20E−04	3.80E−05	1.94E−05	1.74E−05	6.90E−01	2.53E−04	9.24E−07	1.21E−05	− 1.51E−04
5	6.30E−01	1.29E−04	6.48E−05	− 6.75E−05	1.52E−05	5.76E−01	3.53E−04	− 2.93E−06	− 1.22E−05	− 2.82E−04
5	7.16E−01	1.38E−04	7.71E−05	− 5.98E−05	2.85E−05	6.54E−01	3.66E−04	6.62E−06	− 1.30E−06	− 2.68E−04
5	8.02E−01	1.47E−04	8.94E−05	− 5.22E−05	4.17E−05	7.32E−01	3.79E−04	1.62E−05	9.61E−06	− 2.55E−04
5	6.97E−01	1.52E−04	2.10E−05	− 5.87E−07	− 1.75E−05	8.09E−01	3.47E−04	− 2.15E−05	− 5.56E−06	− 2.59E−04
5	7.95E−01	1.66E−04	3.90E−05	1.42E−05	4.31E−06	9.22E−01	3.63E−04	− 1.00E−05	6.27E−06	− 2.43E−04
5	8.93E−01	1.81E−04	5.70E−05	2.91E−05	2.61E−05	1.03E+00	3.79E−04	1.39E−06	1.81E−05	− 2.26E−04
6	1.05E+00	2.15E−04	1.08E−04	− 1.12E−04	2.53E−05	9.60E−01	5.88E−04	− 4.88E−06	− 2.04E−05	− 4.70E−04
6	1.19E+00	2.30E−04	1.29E−04	− 9.97E−05	4.74E−05	1.09E+00	6.10E−04	1.10E−05	− 2.17E−06	− 4.47E−04
6	1.34E+00	2.46E−04	1.49E−04	− 8.69E−05	6.95E−05	1.22E+00	6.32E−04	2.70E−05	1.60E−05	− 4.25E−04
6	1.16E+00	2.53E−04	3.50E−05	− 9.79E−07	− 2.91E−05	1.35E+00	5.78E−04	− 3.58E−05	− 9.27E−06	− 4.32E−04
6	1.33E+00	2.77E−04	6.50E−05	2.37E−05	7.18E−06	1.54E+00	6.05E−04	− 1.67E−05	1.04E−05	− 4.04E−04
6	1.49E+00	3.01E−04	9.50E−05	4.85E−05	4.35E−05	1.72E+00	6.32E−04	2.31E−06	3.02E−05	−3.76E−04

Appliance system	Point A	TD	*X*-axis	*Y*-axis	*Z*-axis	Prosthion	TD	*X*-axis	*Y*-axis	*Z*-axis
1	5.97E−01	1.12E−03	− 4.38E−04	− 7.01E−04	5.55E−04	1.59E+00	1.34E−03	− 4.93E−04	− 8.80E−04	6.18E−04
1	6.62E−01	1.16E−03	− 4.19E−04	− 6.62E−04	6.02E−04	1.80E+00	1.40E−03	− 4.64E−04	− 8.38E−04	6.75E−04
1	7.27E−01	1.20E−03	− 4.00E−04	− 6.22E−04	6.50E−04	2.01E+00	1.45E−03	− 4.34E−04	− 7.96E−04	7.31E−04
1	5.30E−01	1.12E−03	− 4.41E−04	− 7.07E−04	4.97E−04	1.33E+00	1.34E−03	− 5.00E−04	− 8.84E−04	5.79E−04
1	5.88E−01	1.15E−03	− 4.20E−04	− 6.70E−04	5.42E−04	1.51E+00	1.40E−03	− 4.67E−04	− 8.46E−04	6.31E−04
1	6.45E−01	1.18E−03	− 3.99E−04	− 6.32E−04	5.86E−04	1.69E+00	1.45E−03	− 4.34E−04	8.08E−04	6.83E−04
2	8.99E−01	1.22E−03	− 4.90E−04	− 6.10E−04	7.47E−04	2.44175	1.48E−03	− 5.89E−04	− 8.25E−04	8.31E−04
2	9.96E−01	1.27E−03	− 4.60E−04	− 5.57E−04	8.07E−04	2.76E+00	1.55E−03	− 5.55E−04	− 7.68E−04	9.03E−04
2	1.09E+00	1.31E−03	− 4.31E−04	− 5.03E−04	8.66E−04	3.08E+00	1.63E−03	− 5.21E−04	− 7.12E−04	9.74E−04
2	7.99E−01	1.17E−03	− 4.93E−04	− 5.99E−04	6.69E−04	2.03E+00	1.46E−03	− 6.03E−04	− 8.17E−04	7.76E−04
2	8.83E−01	1.21E−03	− 4.62E−04	− 5.55E−04	7.24E−04	2.30E+00	1.53E−03	− 5.62E−04	− 7.67E−04	8.41E−04
2	9.66E−01	1.26E−03	− 4.31E−04	− 5.10E−04	7.78E−04	2.56E+00	1.61E−03	− 5.21E−04	− 7.16E−04	9.07E−04
3	1.49E+00	3.35E−03	− 1.10E−03	− 2.35E−03	1.45E−03	3.97E+00	3.93E−03	− 1.22E−03	− 2.84E−03	1.63E−03
3	1.65E+00	3.45E−03	− 1.05E−03	− 2.24E−03	1.58E−03	4.50E+00	4.08E−03	− 1.14E−03	− 2.73E−03	1.78E−03
3	1.81E+00	3.54E−03	− 1.00E−03	− 2.13E−03	1.71E−03	5.02E+00	0.004226	− 1.07E−03	− 2.61E−03	1.93E−03
3	1.32E+00	3.35E−03	− 1.10E−03	− 2.37E−03	1.31E−03	3.33E+00	3.93E−03	1.24E−03	− 2.85E−03	1.53E−03
3	1.46E+00	3.44E−03	− 1.05E−03	− 2.27E−03	1.43E−03	3.77E+00	4.08E−03	1.15E−03	− 2.75E−03	1.67E−03
3	1.61E+00	3.53E−03	− 9.92E−04	− 2.16E−03	1.55E−03	4.20E+00	4.23E−03	1.07E−03	− 2.65E−03	1.81E−03
4	9.25E−01	2.30E−04	− 2.73E−05	4.74E−05	− 1.77E−04	8.16E−01	2.25E−04	− 6.05E−06	5.57E−05	− 1.85E−04
4	1.05E+00	2.38E−04	− 1.58E−05	5.39E−05	− 1.67E−04	9.21E−01	2.33E−04	− 1.03E−06	6.18E−05	− 1.78E−04
4	1.18E+00	2.46E−04	− 4.34E−06	6.04E−05	− 1.57E−04	1.03E+00	2.40E−04	3.99E−06	6.79E−05	− 1.71E−04
4	8.70E−01	2.36E−04	3.12E−05	4.87E−05	− 1.83E−04	7.32E−01	2.29E−04	9.13E−06	5.91E−05	− 1.86E−04
4	9.85E−01	2.46E−04	4.02E−05	5.57E−05	− 1.74E−04	8.26E−01	2.37E−04	1.37E−05	6.57E−05	− 1.78E−04
4	1.10E+00	2.55E−04	4.92E−05	6.27E−05	− 1.65E−04	9.19E−01	2.46E−04	1.84E−05	7.23E−05	− 1.70E−04
5	1.39E+00	3.45E−04	− 4.10E−05	7.11E−05	− 2.65E−04	1.22E+00	3.38E−04	− 9.08E−06	8.35E−05	− 2.78E−04
5	1.58E+00	3.57E−04	− 2.38E−05	8.09E−05	− 2.50E−04	1.38E+00	3.49E−04	− 1.55E−06	9.27E−05	− 2.67E−04
5	1.77E+00	3.69E−04	− 6.51E−06	9.07E−05	− 2.36E−04	1.54E+00	3.61E−04	5.99E−06	1.02E−04	− 2.56E−04
5	1.31E+00	3.54E−04	4.68E−05	7.31E−05	− 2.75E−04	1.10E+00	3.44E−04	1.37E−05	8.87E−05	− 2.79E−04
5	1.48E+00	3.68E−04	6.03E−05	8.36E−05	− 2.61E−04	1.24E+00	3.56E−04	2.06E−05	9.85E−05	− 2.67E−04
5	1.65E+00	3.82E−04	7.38E−05	9.40E−05	− 2.47E−04	1.38E+00	3.68E−04	2.75E−05	1.08E−04	− 2.56E−04
6	2,31,339	5.74E−04	− 6.83E−05	1.18E−04	− 4.41E−04	2.04E+00	5.63E−04	− 1.51E−05	1.39E−04	− 4.63E−04
6	2.63E+00	5.95E−04	− 3.96E−05	1.35E−04	− 4.17E−04	2.30E+00	5.82E−04	− 2.58E−06	1.54E−04	− 4.45E−04
6	2.94E+00	6.15E−04	− 1.08E−05	1.51E−04	− 3.93E−04	2.57E+00	6.01E−04	9.98E−06	1.70E−04	− 4.27E−04
6	2.18E+00	5.91E−04	7.80E−05	1.22E−04	− 4.58E−04	1.83E+00	5.73E−04	2.28E−05	1.48E−04	− 4.64E−04
6	2.46E+00	6.14E−04	1.00E−04	1.39E−04	− 4.35E−04	2.06E+00	5.93E−04	3.44E−05	1.64E−04	− 4.45E−04
6	2.75E+00	6.37E−04	1.23E−04	1.57E−04	− 4.11E−04	2.30E+00	6.14E−04	4.59E−05	1.81E−04	− 4.26E−04

Appliance system	Maxillary process of zygoma	TD	*X*-axis	*Y*-axis	*Z*-axis
1	3.24E+00	1.09E−03	− 1.37E−04	7.52E−04	6.89E−04
1	3.65E+00	1.19E−03	− 9.33E−05	8.86E−04	7.47E−04
1	4.05E+00	1.30E−03	− 4.93E−05	1.02E−03	8.05E−04
1	3.07E+00	5.40E−04	3.18E−04	2.44E−04	2.57E−04
1	3.38E+00	5.96E−04	3.56E−04	3.68E−04	3.06E−04
1	3.69E+00	6.52E−04	3.94E−04	4.91E−04	3.54E−04
2	4.83E+00	1.67E−03	− 1.95E−04	1.26E−03	9.81E−04
2	5.42E+00	1.83E−03	− 1.32E−04	1.45E−03	1.06E−03
2	6.02E+00	1.98E−03	− 6.85E−05	1.63E−03	1.14E−03
2	4.52E+00	1.08E−03	5.54E−04	7.81E−04	3.91E−04
2	4.97E+00	1.20E−03	6.11E−04	9.53E−04	4.55E−04
2	5.43E+00	1.32E−03	6.68E−04	1.12E−03	5.18E−04
3	8.18E+00	2.49E−03	− 3.05E−04	1.50E−03	1.66E−03
3	9.20E+00	2.71E−03	− 1.94E−04	1.85E−03	1.81E−03
3	1.02E+01	2.93E−03	− 8.32E−05	2.20E−03	1.96E−03
3	7,73,533	1.80E−03	8.16E−04	1.37E−04	6.72E−04
3	8.52E+00	1.98E−03	9.12E−04	4.59E−04	8.00E−04
3	9.31E+00	2.15E−03	1.01E−03	7.81E−04	9.27E−04
4	6.37E−01	2.54E−04	6.87E−05	6.51E−05	2.36E−04
4	6.94E−01	2.72E−04	7.88E−05	7.93E−05	2.55E−04
4	7.52E−01	2.90E−04	8.89E−05	9.36E−01	2.74E−04
4	5.85E−01	2.51E−04	− 4.40E−05	7.79E−05	2.26E−04
4	6.54E−01	2.67E−04	− 3.52E−05	9.36E−05	2.44E−04
4	7.24E−01	2.83E−04	− 2.63E−01	1.09E−04	2.61E−04
5	9.55E−01	3.80E−04	1.03E−04	9.77E−05	3.54E−04
5	1.04E+00	4.07E−04	1.18E−04	1.19E−04	3.83E−04
5	1.13E+00	4.35E−04	1.33E−04	1.40E−04	4.12E−04
5	8.77E−01	3.76E−04	− 6.59E−05	1.17E−04	3.39E−04
5	9.82E−01	4.00E−04	− 5.27E−05	1.40E−04	3.66E−04
5	1.09E+00	4.25E−04	− 3.95E−05	1.64E−04	3.92E−04
6	1.59E+00	6.34E−04	1.72E−04	1.63E−04	5.91E−04
6	1.74E+00	6.79E−04	1.97E−04	1.98E−04	6.38E−04
6	1.88E+00	7.24E−04	2.22E−04	2.34E−04	6.86E−04
6	1.46E+00	6.27E−04	− 1.10E−04	1.95E−04	5.65E−04
6	1,63,588	6.67E−04	− 8.79E−05	2.34E−04	6.09E−04
6	1.81E+00	7.08E−04	− 6.59E−05	2.73E−04	6.53E−04

*Average values of the right side and left side of each suture and the surface landmarks are taken. Appliance system 1—Miniplate—200 g of force, Appliance system 2—Miniplate—300 g of force, Appliance system 3—Miniplate—500 g of force, Appliance system 4—Mini-implant—200 g of force, Appliance system 5—Mini-implant—300 g of force, Appliance system 6—Mini-implant—500 g of force, *FM* fronto-maxillary suture, *ZM* zygomatico-maxillary suture, *ZT* zygomatico-temporal suture, *ZF* zygomatico-frontal suture, *PM* pterygo-maxillary suture, *TD* total displacement, *ANS* anterior nasal spine

### Stress distribution and displacement pattern in craniofacial structures

In the current study, maximum von mises stresses expressed by the maxillary bone was found to be similar in both the groups with respect to all the force levels. However, in miniplates, 500 g of force displayed greater displacement of the maxillary bone. The maximum principal stresses were encountered with the miniplates with the force value of 500 g. The principal minimum stresses and compressive stresses portrays no difference with any of the force systems.

There was a higher stress distribution, principal maximum stresses and principal minimum stress for miniplates especially with 500 g of force in the maxillary dentition, periodontal ligament and glenoid fossa. The maximum stress distribution and displacement was at 500 g of force in all the sutures and anatomical landmarks for both groups. Nevertheless, miniplates with 500 g of force showed highest stress distribution and displacement.

In FM, mini-implant group with 500 g of force was still lesser than the miniplate group even with the lowest force of 200 g. In ZT and frontal process of maxilla, miniplates with 200 g and 300 g also show significant stress distribution. 300 g of force on ZF and 200 g in PM applied using miniplates depict significant displacement of the sutures when compared to the mini-implants.

Maximum stress distribution and displacement in the ANS, point A and prosthion are seen with the miniplates and mini-implants during application of 500 g of force and in miniplate with 200 g and 300 g also. In the maxillary process of zygoma, only miniplate with 300 g of force produces significant displacement. Miniplate with 300 g also produce significant stress distribution at prosthion. At the FA points, the maximum stress distribution occurred with 500 g of force in both the models.

At the root apices, maximum displacement during 200 g and 300 g of force application in the sagittal plane occurred with the central incisor and lateral incisor respectively. During 500 g of force application, first premolar and first molar experienced maximum displacement. In the transverse plane, second premolar and first molar experienced significant root displacement in all three force systems with miniplate while with the mini-implants, canine shows maximum lingual root displacement with the application of 200 g and 300 g of force and lateral incisor shows maximum root displacement with 500 g of force. In the vertical plane, canines showed maximum displacement during 200 g of force application and during 300 g and 500 g of force application, central incisors showed maximum intrusion with miniplate. With mini-implants, central incisors shows maximum intrusion during application of all three types of forces.

## Discussion

When employing distalization appliances, the untoward effects on the anteriors have been inevitable responses to the distalizing force [17, 18]. Hence, temporary anchorage devices are considered. The most popular movement carried out using the temporary anchorage devices were distalization and intrusion of the dentition [19]. In the model 1 of the current study, miniplates were fixed to the zygomatic buttress in order to facilitate intrusion and distalization.

Bechtold et al. determined that greater distalization and intrusion effects can be achieved with dual miniscrews rather than a single miniscrew [15]. Hence, the model 2 of the current study employs two mini-implants. Rudolph et al. determined the types of orthodontic forces which cause high stress at the root apex and concluded that for intrusion, extrusion and rotational forces, there was an increased stress concentration at the root apex [20]. Hence, in the current study, root apices were also inspected in order to elucidate the displacement tendency.

### Stress distribution and displacement pattern

#### A) Maxillary dentition, PDL and glenoid fossa

In the maxillary dentition, the displacement had occurred maximally in the vertical and sagittal directions with maximum stress concentration and displacement at the lateral incisors and canine. Eui-Hyang Sung et al. explains that the reason behind the pattern of displacement is the presence of the retraction hook in the area which holds good for the current study also [21].

In the PDL, mini-implants displayed a higher stress distribution in comparison with the miniplates. This could be attributed to the site of placement of mini-implant being between the inter-dental bone. Displacement was evident majorly in the vertical direction.

The glenoid fossa shows maximum displacement in the vertical direction followed by the sagittal direction thereby facilitating intrusion and distalization.

Calcada et al. determined the stress on TMJ during chincup therapy and determined that only a minimal amount of a stress is distributed over the glenoid fossa which is in correlation to the current study [22].

#### B) Craniofacial sutures

Py Owman-Moll et al. suggested that there is a 50% increase in the stress levels when the orthodontic forces increase by four-fold [23]. Hence, this study employs three different force levels. Lee and Baek determined that the stresses seen at the frontomaxillary, zygomaticomaxillary and pterygo-maxillary sutures were increased with miniplates placed at the infrazygomatic crest [24].

In the frontomaxillary suture, displacement occurred majorly in the vertical plane causing compression and a more uniform stress distribution which is in accordance with a study by Holberg et al. [25]. In the zygomatico-maxillary suture, sagittal plane displacement was predominant depicting the influence of distalization forces which is concurrent with Pawan Gautam et al. [16]. In the zygomatico-temporal suture, distinct patterns of bone strain was depicted by Oberheim and Mao during headgear therapy indicating a differential response of the sutures [26]. Von mises stresses are the greatest for the zygomatico-temporal suture which is in accordance with a study by Gautam et al. [16]. In the zygomatico-frontal suture, displacement occurred majorly in the vertical plane which represent the effect of intrusive forces which causes compression over the suture. Yan et al. determined that during protraction of the maxillary arch, there is an stress distribution with the pterygomaxillary suture [27]. The current study also portrays a similar pattern along with displacement pattern in the sagittal direction.

In the frontal process of maxilla, an increased displacement in the vertical plane was seen. An increased stress was elucidated which complies with Seong et al. [28]. In the anterior nasal spine, increased intrusive effect could be identified along with an upward and backward movement of the ANS. At point A, with 500 g of force, there is a maximum displacement and a posterior and upward movement of because of the intrusive and distallizing force which is similar to Lee et al. [24]. At the prosthion, an upward and backward displacement is elucidated. At the maxillary process of zygoma, vertical plane displacement is higher with more intrusive effects rather than a transverse movement.

#### C) Facial axis point

**Fig. 5:**
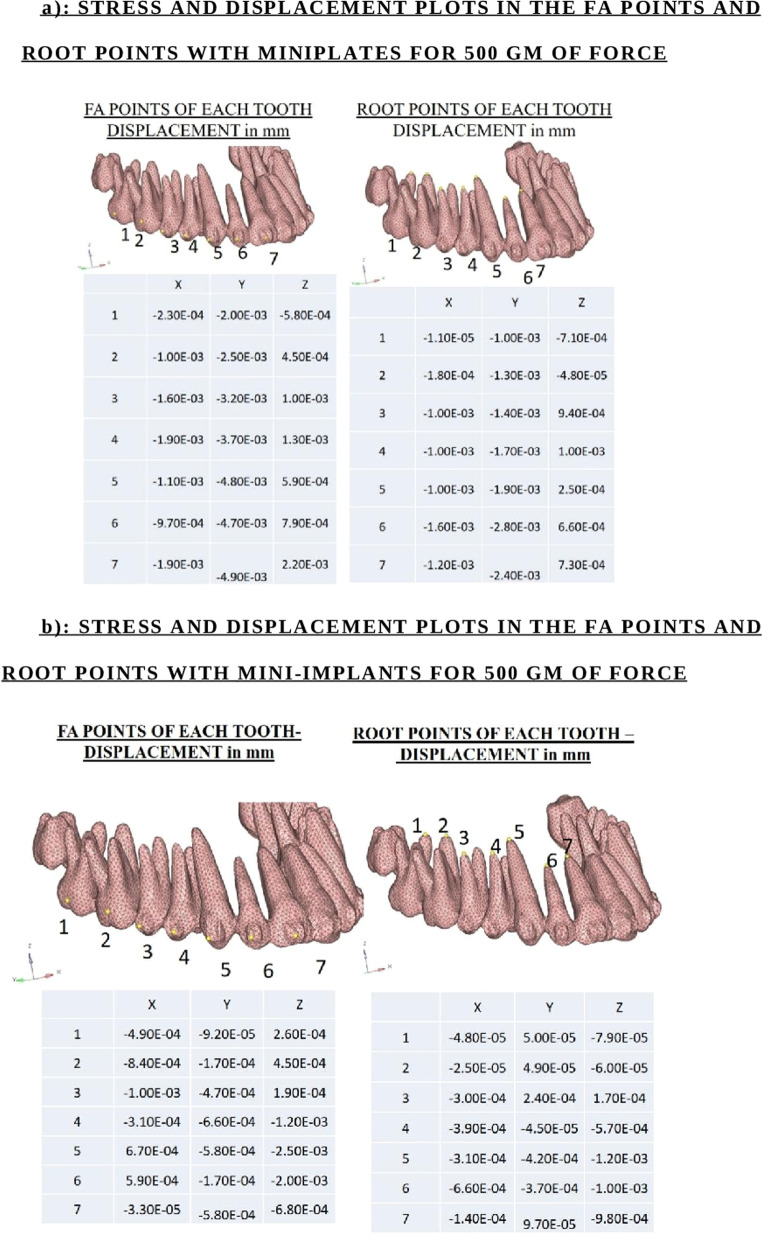
Stress and displacement plots in the FA points and root points for 500 gm of force. **a** With miniplates. **b** With mini-implants

In the sagittal direction, maximum stress was at the first molar during 500 g of force application indicating distalization effect of the miniplates (Fig. 5a). In the transverse direction, there was an increased displacement in the first and second molar indicating a buccal tipping of the molar because of the point of force application being posterior to the segment as suggested by Sung et al. [21]. In the vertical plane, maximum displacement occurred with the central incisors indicating maximum intrusion.

In mini-implant, sagittal plane, the first molar and first premolar shows maximum displacement indicating distalization (Fig. 5b). In the transverse plane, first premolar, first molar and canine show significant lingual rolling. In the vertical plane, central incisors and canine show maximum displacement indicating intrusion.

On comparing the groups, greater sagittal movement is obtained with Model 1 especially with 500 g of force application with increased buccal flaring in the transverse plane. In the vertical plane, Model 2 showed greater intrusion with significant intrusion of the canine.

#### D) Root apices

During 300 g and 500 g of force application, central incisors showed maximum intrusion. In the mini-implant model, with 500 g of force, the lateral incisor shows maximum root displacement which is in similarity to Sung et al. [21]. In the vertical plane, central incisor shows maximum intrusion during application of all three types of forces.

## Conclusion

### Stress distribution

1. Miniplates show higher stress distribution with highest stress at the zygomatic buttress region.
2. In the PDL, mini-implants had higher stresses.
3. The glenoid fossa shows greater stress distribution with the miniplates.
4. Miniplates show significant stress distribution in the fronto-maxillary, zygomatico-temporal, zygomatico-frontal and pterygomaxillary sutures, frontal process of maxilla, anterior nasal spine, Point A, prosthion and maxillary process of zygoma.

### Displacement pattern

1. Miniplates show greater displacement patterns with 500 g of force.
2. In the PDL, mini-implants showed greater stress distribution majorly in the vertical direction.
3. In the glenoid fossa, the displacement occurred in the sagittal and vertical direction.
4. Fronto-maxillary, zygomatico-temporal, zygomatico-frontal sutures, frontal process of maxilla and the maxillary process of zygoma experiences majorly a vertical force.
5. Zygomatico-maxillary and pterygomaxillary sutures experiences predominantly a sagittal displacement.
6. With miniplates, first molars experience greater distallisation while with mini-implants, canine and premolars also exhibit greater distallisation effects.
7. Central incisors and lateral incisors experienced maximum intrusive effects in both groups. However, the mini-implants produce greater intrusive effects.
8. In the root apices, lateral incisors show increased lingual root movement with mini-implants.

**Fig. 6:**
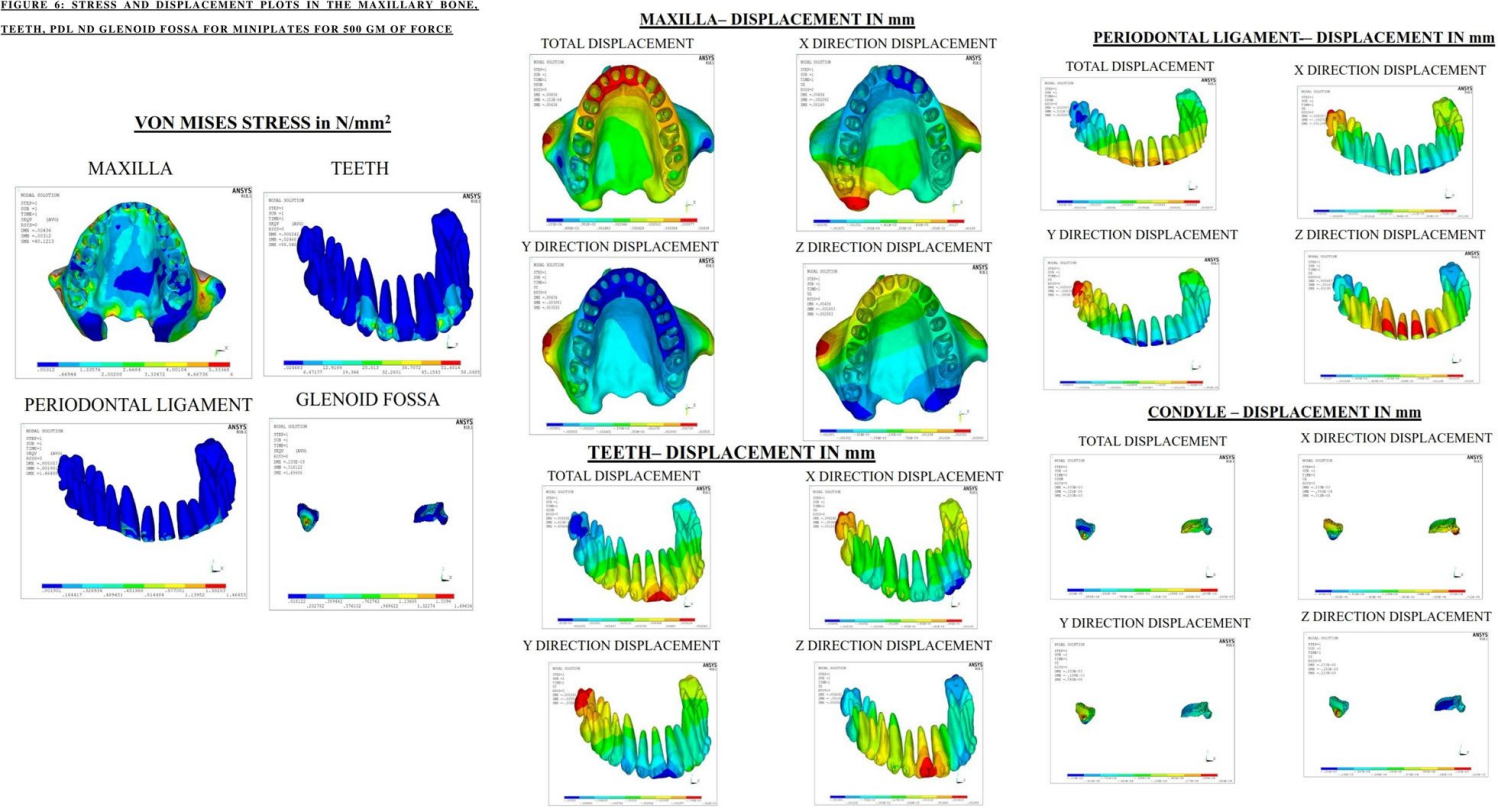
Stress and displacement plots in maxillary bone, teeth, PDL and glenoid fossa with miniplates for 500 g of force

**Fig. 7:**
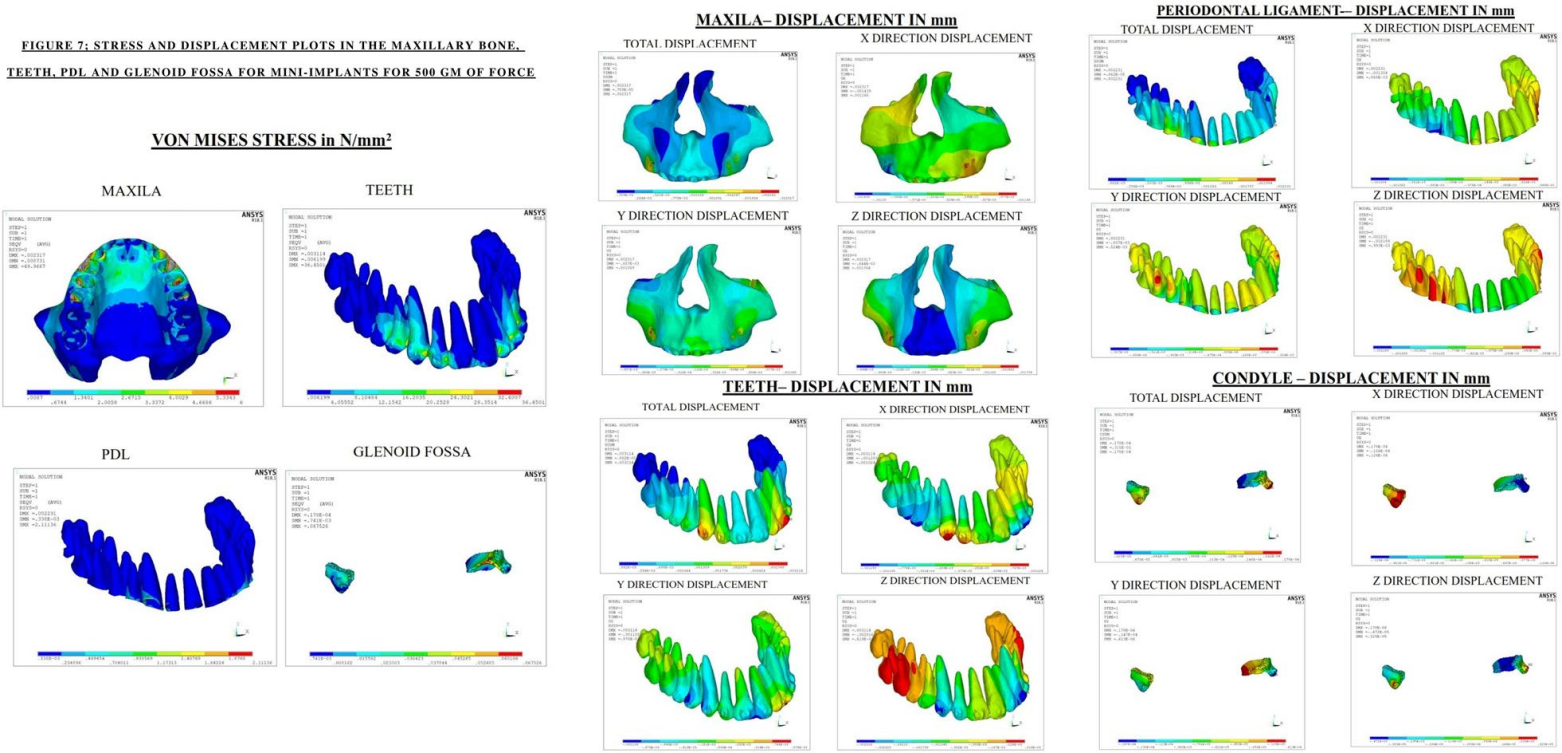
Stress and displacement plots in maxillary bone, teeth, PDL ND glenoid fossa with mini-implants for 500 g of force

In toto, comparing both groups, miniplates produce a greater distallising effect while the mini-implants produce increased intrusive effect. The distallising effect is greater with 500 g of force using miniplates with significant stress distribution and displacement pattern (Figs. 6, 7).

## Data Availability

Data will be shared if needed.
